# Crawling Cells Can Close Wounds without Purse Strings or Signaling

**DOI:** 10.1371/journal.pcbi.1002007

**Published:** 2011-03-10

**Authors:** Pilhwa Lee, Charles W. Wolgemuth

**Affiliations:** University of Connecticut Health Center, Department of Cell Biology and Center for Cell Analysis and Modeling, Farmington, Connecticut, United States of America; University of California, San Diego, United States of America

## Abstract

When a gash or gouge is made in a confluent layer of epithelial cells, the cells move to fill in the “wound.” In some cases, such as in wounded embryonic chick wing buds, the movement of the cells is driven by cortical actin contraction (i.e., a purse string mechanism). In adult tissue, though, cells apparently crawl to close wounds. At the single cell level, this crawling is driven by the dynamics of the cell's actin cytoskeleton, which is regulated by a complex biochemical network, and cell signaling has been proposed to play a significant role in directing cells to move into the denuded area. However, wounds made in monolayers of Madin-Darby canine kidney (MDCK) cells still close even when a row of cells is deactivated at the periphery of the wound, and recent experiments show complex, highly-correlated cellular motions that extend tens of cell lengths away from the boundary. These experiments suggest a dominant role for mechanics in wound healing. Here we present a biophysical description of the collective migration of epithelial cells during wound healing based on the basic motility of single cells and cell-cell interactions. This model quantitatively captures the dynamics of wound closure and reproduces the complex cellular flows that are observed. These results suggest that wound healing is predominantly a mechanical process that is modified, but not produced, by cell-cell signaling.

## Author Summary

Wound healing is driven by the collective migration of groups of epithelial cells. Experiments have shown that the motions of cells during wound healing are not as simple as had once been thought. Indeed, cells do not just move out to fill in the wounded area but rather undergo a number of complex but coordinated motions. Furthermore, wound healing is not just a response to chemical cues and can be driven by cells that are not immediately at the edge of the wound. In this paper, we develop a mathematical model based on the mechanical behavior of single crawling cells and also includes cell-cell adhesion. We show that this model is capable of explaining quantitatively the dynamics that occur during wound healing assays. This suggests that wound healing is largely a mechanical process where chemical signaling merely acts to augment the overall behavior.

## Introduction

One important feature of embryonic and organ development is the collective migration of groups of cells [1]–[4]. In some cases during development, large groups of cells move in streams with each cell independently following chemotactic cues from the environment [5]. However, during the morphogenesis of organ systems, wound healing, and cancer metastasis, it is more common to find cells migrating as an adherent group [4]. Some examples of these motions are the movement of cells during the morphogenesis of the inner blastocyst [6], epithelial cell migration at the rim of the optic and the invaginating thyroid placode [7], tissue repair by keratinocytes moving across provisional wound matrix [8], and the migration of border cells through the early ovary in *Drosophila* [9]. Though the fundamental features of single cell motility are now understood at some level [10]–[12], the physical underpinnings of the collective migration of groups of cells remains enigmatic.

In this paper we focus on understanding the collective migration of epithelial cells during wound healing. Two separate mechanisms have been proposed to account for wound closure. In the first, a circumferential ring of actin bundles contract to draw the wound edges together [13]. This mechanism has been demonstrated in embryonic chick wing buds; however, wound closure in adults is presumed to rely on the crawling motility of epithelial cells [13], [14]. The cellular mechanics of this latter form of wound healing remains unclear. We propose that the fundamental driving force behind this process is the generic migratory behavior of an individual cell, and, specifically, that the active contractile stress generated within polarized epithelial cells coupled with cell-cell adhesion is sufficient to explain many of the features observed during wound healing assays.

Though there is significant variation in the biochemical composition of different crawling cells, the basic biophysical process of single cell crawling entails (i) cytoskeletal extension at the front of the cell; (ii) adhesion to the substrate, which is typically mediated by integrin; and (iii) advance of the rear [10]–[12]. In addition to these fundamental features, crawling cells are almost always polarized [15], [16] and are observed to exert a dipole-distributed traction stress on the substrate [17]–[20]. Neighboring cells can bind to one another through membrane-bound cadherin molecules [21], [22]. We hypothesize that bulk cellular motions in tissue are strongly dependent on these general features and that other specific details of single cell crawling are less important.

In wound healing assays, a gouge is made in a continuous monolayer of cells (often MDCK cells are used), and the rate that the cells fill in the artificial wound is measured. There are a number of features that are observed in these assays that suggest that the healing process is not solely reliant on biochemical signaling triggering the migration of cells into the denuded area. The cells in the wounded monolayer typically migrate in groups and maintain cell-cell contacts [23]–[25]. Cells many cell diameters away from the wound edge are motile [23]–[25], and the rate of migration away from the edge is observed to be inversely proportional to the distance from the margin [25]. At the wound edge, cells do not always migrate perpendicular to the boundary [24], [26], and cell division is not observed to play a strong role in closing the wound [24], [25]. Interestingly, it is also observed that the wound border progression advances roughly proportional to time squared [24], [27]; i.e., the average boundary velocity increases proportional to time since wounding.

In this study, we explore the possibility that the collective cellular migration that occurs during wound healing is largely a mechanical process. We develop a mathematical model that incorporates the bulk features of single migrating cells and cell-cell adhesions. We first apply the model to the average motion of a spreading strip of cells and identify the key physics that may drive the advance of the cells at the wound edge. We then consider the healing of circularly-symmetric wounds and show that the model can reproduce wound closure when rows of cells at the wound periphery are deactivated. Finally, we simulate two-dimensional wound healing assays and show that our model can reproduce the complex cellular flows and border advance that is observed in experiments. Taken together, these results show that wound healing may not require substantial biochemical signaling or even mechanisms to identify wounding, but rather may result from the typical dynamics of motile cells.

## Results

### A single cell based model for the collective migration of epithelial cells

**Figure 1:**
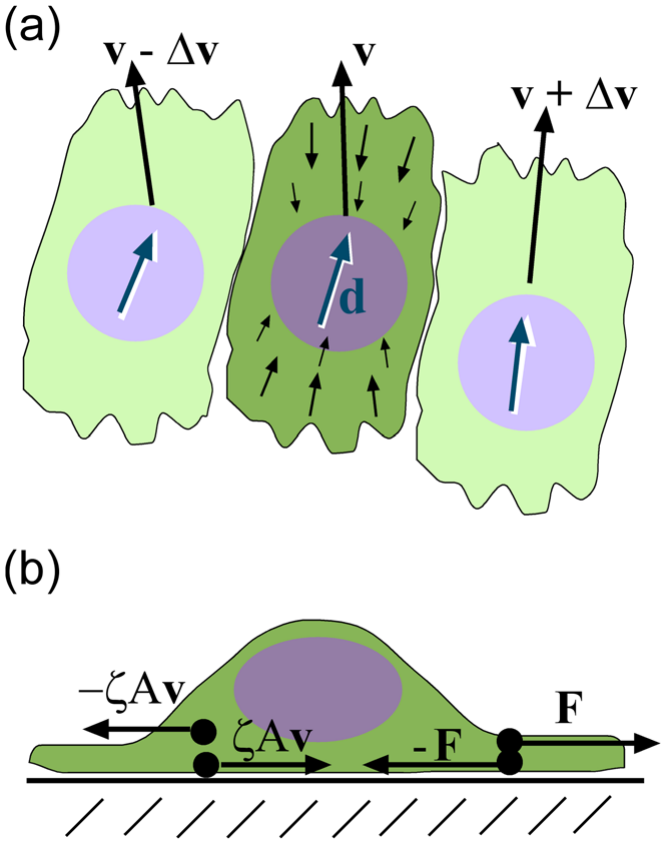
Schematic of the model depicting the cell orientation and forces. (a) Cells are aligned along the direction **d** and move with velocity **v**. Differences in velocity between neighboring cells produce a viscous stress. Neighboring cells preferentially align with differences in the orientation producing a torque on neighboring cells. Cells exert a dipole-distributed stress on the substrate and also on neighboring cells. (b) Each cell exerts a net force -**F** on the substrate. An equal but opposite force is exerted back on the cell. The substrate also exerts a drag force -ζA**v** on each cell. The thrust force and the drag force are offset, which produces the effective dipole stress on the cell.

Inside a crawling eukaryotic cell, the actin cytoskeleton flows rearward at the front of the cell and forward at the rear of the cell [28]. Nascent and/or mature focal adhesions, which include integrin, link the cytoskeleton to the substrate or extracellular matrix (ECM) [29], and thereby convert the cytoskeletal flows into traction stresses that are applied to the substrate [17], [20], [30]. Like the actin velocity, the force that the cell exerts on the substrate is rearward at the front and forward at the rear; i.e., it is distributed like a dipole (**Figure 1**a) shows the traction stress inside a cell that is polarized along the direction d) [17], [20], [30]. These dipole-distributed traction stresses, σ_d,_ lead to a net thrust force F that propels the cell at roughly constant velocity (**Figure 1**b). For example, isolated MDCK cells plated on a substrate spread to be about 20 µm long and crawl at speeds of about 10 µm/hr [23]; the magnitude of the traction stress that the cell exerts on the substrate is of order 3×10^4^ dynes/cm^2^ [30]. The turnover rate of integrin inside focal adhesions is on order of a minute [31], and, therefore, integrin turnover is fast compared to the crawling speed of the cell, which allows us to treat the interaction between the cytoskeletal flows and the substrate as a resistive drag force that is proportional to the velocity, with drag coefficient ζ [32].

Epithelial cells that are in close contact can adhere to one another through cadherin molecules [21], [22]. The turnover of cadherin molecules in cell-cell adhesions is on the order of tens of minutes to an hour, which is significantly slower than the turnover rate of integrin in focal adhesions [22]. For timescales less than this turnover time τ, neighboring cells are effectively stuck together. A tissue of cells should therefore behave like an elastic solid on short timescales. On longer timescales, though, cadherin turnover allows the cells to slide with respect to each other, and the bulk tissue should behave more like a fluid with viscosity η. Therefore, the stress between cells is maintained on times shorter than τ, but dissipates on longer timescales. Cells in monolayers overlap [25] and the initial 10–12 hours of the dynamics of wound healing are not dependent on cell division [24], so we do not track the density in our model. We assume, though, that changes in density are resisted by a different effective viscosity than shear displacements and define a volumetric viscosity (λ - η/2). The intercellular stress σ_c_ can then be described with the Maxwell model,
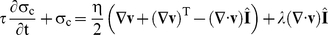
(1)which is a simple model for viscoelastic fluids. In (1), 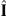 denotes the identity matrix. Our choice of this cell-cell interaction model is justified by analyzing the behavior of two solid objects connected by spring-like adhesion molecules, which is based on a model for muscle cross-bridges developed by Lacker and Peskin [33] (See supplemental **Text S1**B for a complete description of how this model leads to the Maxwell model).

For crawling cells, the resistive drag forces are large compared to the inertial terms. Therefore, the sum of all of the forces acting on a cell must be equal to zero. In our model, we consider four types of forces that act throughout the monolayer. First is the force produced by the intercellular stress that is described above (Eq. 1). The second force is due to the internal stresses that are generated inside single cells. This stress, which we denote by σ_d_, includes the viscoelastic stress of the cytoskeleton, as well as the active stresses from actin dynamics and molecular motors, such as myosin. For our model, we consider that this stress is largely dipole-distributed along the polarization direction of the cell and set it equal to its average value *f*_0_ *b* **dd**, where *f*_0_ is the dipole force and *b* is the dipole length. The actin flow inside a cell interacts with the substrate through adhesions and produces the thrust force **F** against the substrate. Finally, motion of the cell with respect to the substrate is resisted by drag forces, which are also due to the cell-substrate adhesions. We average the internal forces that are generated by a cell and balance these with the average external applied forces on the cell, which provides a mean-field dynamic equation governing the flow of the cells (for complete details, see Text S1A):
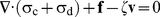
(2)where **f** = **F**/*A* is the thrust force per unit area, and *A* is the area of a cell. In this model, we assume that the magnitude of the thrust force is a constant. The velocity v in Eq. 2 defines the average local velocity of the cells in the monolayer.

To complete the biophysical description of the epithelial cell layer, we must define the dynamics of the cell polarization. We assume that changes in cell orientation are driven by the mechanical interactions between cells as they move in the monolayer. We consider two torques that act to determine cellular orientation. First, the polarization of the cells combined with the cell elasticity favor alignment of neighboring cells (**Figure 1**). When neighboring cells are not aligned, there is a restoring torque that acts to align them. Therefore gradients in the orientation produce an elastic torque similar to the torque on a nematic liquid crystal. For this model, we use a single Franck constant, *K*, to describe the magnitude of the elastic restoring torque. Second, a resistive drag torque impedes the reorientation of the cells and is proportional to the time rate of change of the orientation vector. The re-orientational dynamics are then similar to that for nematic liquid crystals [34]:
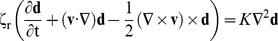
(3)


Here ζ_r_ is a drag coefficient, and v is the velocity field for the cells. The second term on the lefthand side represents changes in orientation due to advection. The third term represents rotation of the polarization due to the motion of the cells (see **Text S1**C for more details). Eqs. 2 and 3 are similar to equations that have been used to describe the collective swimming of bacteria [35]. In these systems, complex flow patterns are observed that are characterized by transient vortices and jets [36].

### Parameter estimation from experimental data

The model that is described in the previous section has a total of 7 parameters (the dipole stress strength, the substrate drag coefficient, the magnitude of the thrust force, the cadherin turnover rate, the viscoelastic shear and volumetric viscosity, and the ratio of the Franck constant to the substrate drag coefficient). Because the model is based on the biophysics of single cell motility, many of these parameters can be estimated from experimental data. The magnitude of the dipole stress is given by the length of the cell (∼10 µm [23]) times the traction stress measured in experiments (∼ 10^4^ dynes/cm^2^ [30]). The traction stress is also equal to the substrate drag coefficient, ζ, times the local cytoskeletal velocity. At the rear of the cell, the velocity is equal to the average single cell crawling speed (10 µm/hr [23]), and, therefore, ζ is approximately 100 pN·hr/µm^3^. At the front of the cell, the retrograde flow rate (1.5 µm/hr [28]) and, therefore, ζ is approximately 500 pN·hr/µm^3^. The average value of ζ should be between these two values. The magnitude of the thrust force, F, is the substrate drag coefficient times the average cell speed multiplied by the area of the cell, which is approximately 10^5^ pN. As mentioned previously, the cadherin turnover rate, τ, is between 15 minutes and 1 hour [22].

Much less is known about the final three parameters in the model. We treat the cell-cell shear viscosity as a free parameter and make the assumption that the volumetric viscosity is about ten to one hundred times larger than this value. To estimate the Franck constant, we use the results from wound healing assays where the polar order parameter, which describes the orientation of the velocity field with respect to the border, was measured as a function of time [26]. In these experiments, it is observed that the order parameter asymptotes to a fixed value in about 20 hours. If we assume that this orientational ordering arises due to the preference for neighboring cells to align, then this timescale should be approximately equal to the elastic relaxation timescale for the orientational dynamics. Therefore, ζ_r_ *L* ^2^/4π^2^ *K* should be approximately 20 hrs. Using the observed velocity correlation lengthscale, *L* ∼ 200 µm [24], [26], we get that *K*/ζ_r_ is 50 µm^2^/hr. If we then assume that the orientational drag coefficient is predominantly due to sliding against the substrate, we expect that ζ_r_ ∼ ζ*b*/24  = 40 pN·hr/µm^2^, and *K* is then approximately 2×10^3^ pN.

### A simplified look at the advance of an initially-straight border

**Figure 2:**
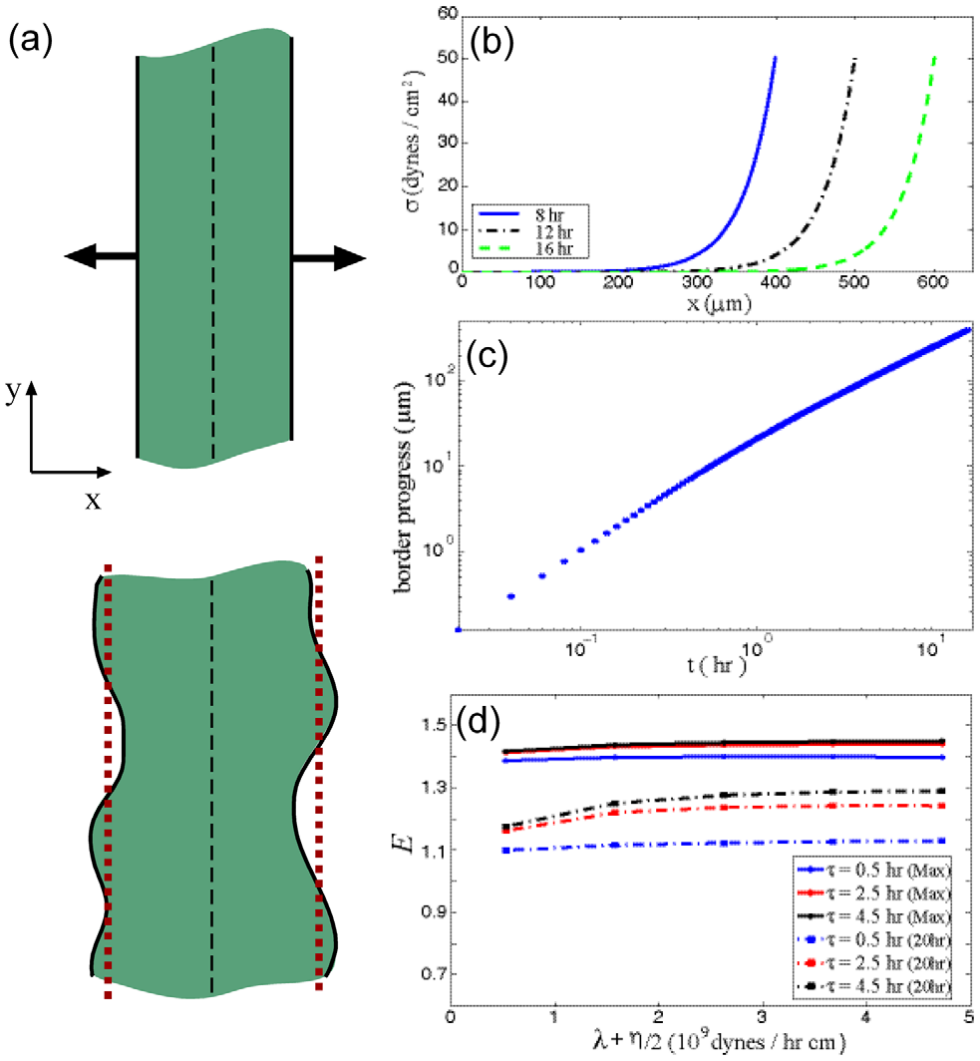
Healing of a one-dimensional wound. (a) Schematic of the 1D model for wound healing. A strip of cells (shown in green) aligned along the y-direction crawls to fill in the denuded area adjacent to them. Though the border advance is irregular, we consider the average advance of the border as a function of time (depicted by the red dashed line). (b) The average stress profile inside the band of cells. The stress is highest at the edge and the profile moves as a travelling front with the motion of the cells. (c) The net advance of the edge as a function of time. (d) The border progress exponent as a function of the viscosity and viscoelastic time constant. As observed in experiments, the border expands nonlinearly with time. At later times (∼20 hrs), the exponent has decreased to closer to one.

In typical wound healing assays, a relatively straight gouge is made through a monolayer of cells, and the rate that the cells fill in the denuded area is measured. To gain insight into the fundamental workings of our model, we consider a long strip of cells with vacant substrate bounding either side of the strip (**Figure 2**a). If we now average the dynamics of the cells over the entire length of the strip, assuming that the cellular orientations are isotropically distributed, then the orientational dynamics (Eq. 3) and the thrust force **F** average to zero. In addition, the net affect of the dipole-distributed stress is an over all compressive pressure that is exerted on to the substrate. An expansive pressure is, therefore, exerted back on the cell strip, and it is this pressure that drives the expanse of the cells into the wounded area.

The averaged dynamics in this simplified system is given by the balance of the forces due to the viscoelasticity of the cell-cell adhesions and the drag with respect to the substrate, driven out at the boundary by the effective dipole-induced pressure. The derivation of the resulting mathematical system is given in **Text S1**D. We solve the one-dimensional model with a free boundary that moves with the average velocity of the cells at the boundary. The 1D equations are integrated using a semi-implicit, finite difference scheme that is described in detail in **Text S1**F. All simulations used a time step of 0.001 hr and solved on a domain with 512 grid points. To compare to experimental data, we compute the net displacement of the boundary as a function of time. In addition, a number of experiments have observed that the net displacement of the border advances roughly proportional to time squared, when the width of the cell strip is larger than 200 µm [24], [27]. This “acceleration” of the border is somewhat counter-intuitive, as random cellular motions should lead to diffusive behavior that scales like the square root of time, and if the cells crawl at constant velocity, then one would expect the border advance to scale linearly with time. Interestingly, for cell strips with initial widths less than 200 µm, the border advance does scale roughly proportional to time [24]. In order to explore whether our model can explain this interesting bi-phasic behavior, we also compute the exponent of the time dependence of the border progression, *E*, as a function of time.

In general, the model produces a uniformly distributed stress away from the boundary of the cell strip, with a sharp transition at the boundary, which reflects the stress generated by the expansive pressure. This stress profile is stable and moves along with the boundary, similar to a traveling front (Figure 2b). In this simplified system, the model depends on two parameters, the cadherin turnover rate τ and the volumetric viscosity (λ+η/2). When the turnover rate is fast or the viscosity is high, then the transition region of the stress is larger than when the turnover rate is slower or the viscosity is lower.

We find that the border progression exponent is greater than one (i.e., the border advances supra-linearly in time), regardless of our choice of parameters; however, for times over about 20 hours, the exponent asymptotes to one (**Figure 2**c). During the first ten hours, the exponent is maximal. The value of the maximum exponent and the value at 16 hours are shown in **Figure 2**c. The cadherin turnover time strongly influences the intermediate value of the border progression exponent, with larger turnover times increasing the exponent. The exponent *E* is not influenced strongly by the cell-cell viscosity, as the viscosity determines the overall speed of the cellular flow, but does not limit the expansion at early times (**Figure 2**c). We also find that the maximal border progression exponent is not dependent on the initial width of the cell strip.

### The healing of circular wounds

Though a standard wound healing assay makes a long, relatively straight gouge through an intact monolayer of cells, many wounds in vivo are more localized and have a circular or ellipsoidal geometry. In fact, these roughly circular-shaped wounds can allow actin contraction at the wound periphery to close the wound (i.e., the purse-string mechanism [13]). Wounds with these geometries can be made in vitro by scraping a micro-injection needle over the surface using a micro-positioner [14]. MDCK cell monolayers wounded in this fashion with an ellipsoidal-shaped wound with minor axis of ∼100 µm close in approximately 10 hours [14]. Interestingly, inhibiting Rac, a protein that is associated with formation of lamellipodia, in a single row of cells at the wound edge does not stall wound closure; however, inhibiting Rac in the first three rows does. Therefore, the purse-string mechanism is not required for wound healing in this system, and the crawling motility of cells away from the wound edge can drive wound closure.

**Figure 3:**
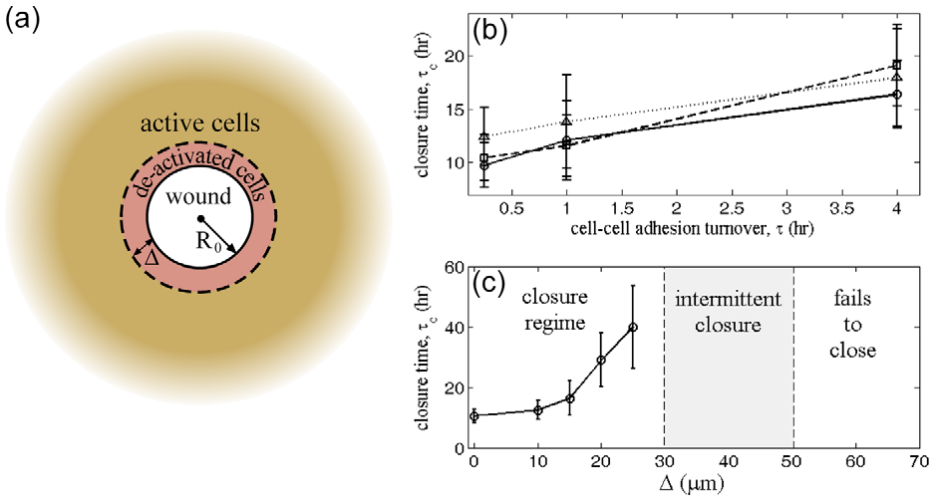
Healing circular wounds. (a) Schematic of a circular wound. A circular-shaped wound with radius R_0_ is made in an intact cell monolayer. In a small region of size Δ about the wound, cellular actin dynamics is deactivated by Rac inhibition. The time to close the wound, τ_c_, can be predicted from the model. (b) When Δ = 0, the model predicts that the closure time is strongly dependent on the viscoelastic timescale τ, which is set by the turnover rate of cell-cell adhesions. The Franck constant *K*, which defines the preference for neighboring cells to align, does not produce a statistically significant effect on the closure time (*K* = 0.064 (solid line), *K* = 0.256 (dashed line), and *K* = 1.024 (dotted line)). (c) Deactivation of the cells in a small region about the wound boundary leads to an increase in the closure time. For deactivation zones that are between 30–50 µm, we find that the wounds close intermittently. Above 50 µm, the wounds consistently fail to close. Error bars show one standard deviation in the closure time from simulations that were started with random initial conditions.

To test whether our model can reproduce these findings, we consider a circular-shaped wound with an initial radius, R_0_, which we set to be 100 µm (**Figure 3**a). By averaging the equations about the circumferential direction, we can solve a one-dimensional model that describes the average closure dynamics of this circular wound (See **Text S1**E for the mathematical details). The equations are integrated using a semi-implicit, finite difference scheme that is described in detail in **Text S1**F. All simulations used a time step of 0.001 hr and solved on a domain with 512 grid points. We use a random initial condition, and determine how the closure time τ_c_ depends on the model parameters. We find that the parameter that influences the closure time the most is the viscoelastic time constant τ (**Figure 3**b), with larger values of τ leading to longer closure times. Varying the Franck constant *K* does not produce a statistically significant effect on the closure time (**Figure 3**b), and neither does varying the viscosities η and λ.

In our model, we can also “de-activate” the actin dynamics in a band of cells that borders the wound by setting the thrust force and dipole stress terms to zero. We define a region of size Δ about the wound edge, where the actin dynamics are deactivated. By varying the width of this region, we simulate the closure of the wound and measure the average closure time as a function of the width. For widths of the deactivated zone up to 0.3 R_0_, we find that the average closure time increases (**Figure 3**c). For widths between 0.3–0.5 R_0_, it is not possible to define an average closure time, as the wounds do not close for all initial conditions. We define this as the intermittent closure regime. When the width is above 0.5 R_0_, we find that the wound always fails to close. Since typical experiments examined wounds that were of order of 100 µm, our model is in good agreement with the observation that deactivation of 3 rows of cells prevents wound closure.

### The two-dimensional dynamics of a wounded epithelial monolayer

In the preceding sections, we have shown that our model can capture the motion of the boundary that accompanies wound healing for simple geometries. These simulations allow us to determine reasonable values of the unknown model parameters, such as the Franck constant and the effective viscosities that are due to cell-cell adhesion. Experiments that monitor the motion of cells in the two-dimensional plane of the substrate are able to visualize the complex flows of cells that accompany wound healing and the traction stresses that are exerted on the substrate during wound closure. As has been already mentioned, these experiments show vortical motion of the cells in the monolayer with long-range correlations in the velocity field over lengths of roughly 100 µm [26], fingering at the wound boundary, supra-linear advance of the boundary with respect to time with an exponent that depends on the initial width of the epithelial monolayer [24], and high traction stress in the immediate vicinity of the boundary [30].

**Figure 4:**
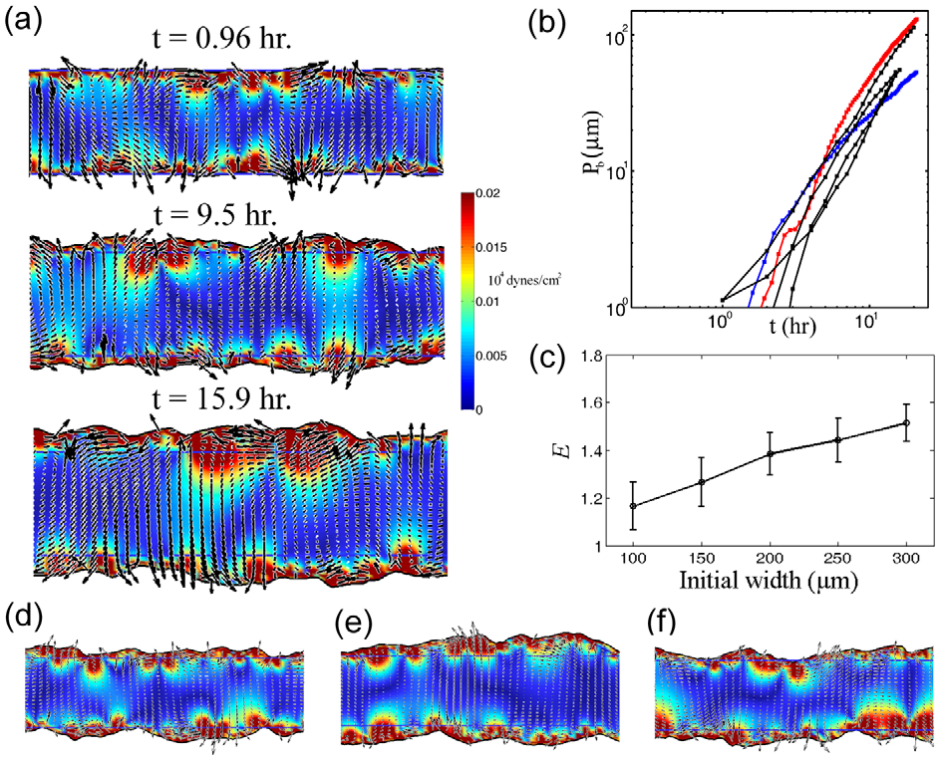
Complex flows and border progression in two-dimensional wound healing assays. (a) A characteristic time course from one of our simulations with an initial width of 200 µm showing the local velocity of the cells (black arrows) and the traction force exerted against the substrate (colormap). Inside the cell-filled region, the cells move with complex dynamics, which include vortices and long-range correlations in the velocity field. The border advance is non-uniform and shows characteristics of a fingering-type instability. (b) The average advance of the border matches well data from experiments by Poujade, et al. [24] when the initial width of the cell-filled region is above 200 µm (red points). Also shown is a simulation with an initial width of 100 µm (blue points). As seen in experiments, smaller initial widths lead to a smaller exponent for the advance of the border. (c) The border advance exponent *E* increases with the initial width of the cell-filled region. The error bars show one standard deviation (*N* = 10). (d)–(f) Other characteristic internal flows and border shapes from simulations, highlighting the appearance of vortices and border fingering. All simulations use an initial width of 200 µm (horizontal blue lines). These simulations use the parameter values given in **Table 1**.

Using the force and stress parameters that were estimated from experiments and the Franck constant and viscosities that were determined from our 1D simulations (see **Table 1**), we solve the two-dimensional, free boundary problem of an infinite strip of cells with initial width *L*_0_. The dynamic equations were discretized and solved using the Moving Boundary Node Method [37]. This method is a level set-based, finite volume algorithm (further details of the numerical routine are given in **Text S1**). For these simulations, we used a time step of 0.001 hr and a grid spacing that was 1/40 of the initial width of the domain (i.e., for a monolayer with an initial width of 300 µm, the grid spacing was 7.5 µm). We initialize our simulations with zero stress and a random orientational field. We used multiple simulations to explore the variation that is caused by the random initial condition. These simulations show many of the features observed in the experiments. For example, the motion of the cells is spatially nonuniform; however, there exists long-range correlations in the velocity field over distances of 100–200 µm (**Figure 4**a). Transient vortices are also observed. Near the boundary the cells do not always move perpendicular to the boundary and the boundary shows characteristics of a fingering instability (**Figure 4**a, d–f). However, the fingering of the border that we observe in our simulations is not as pronounced as is sometimes observed in experiments. As in the 1D simulations, there is an increase in the traction stress that is exerted on the substrate, and this increased stress dies off within 10–20 µm from the boundary (see colorscale in (**Figure 4**a, d–f).

**Table 1:** Model Parameters.

Parameter	Symbol	Value	Source
Viscoelastic time scale	τ	0.25 hr	E.E.* [22]
Effective shear viscosity	η	10 dynes×hr/cm	E.S.†
Volumetric viscosity	λ	10^3^ dynes×hr/cm	E.S.†
Substrate drag coefficient	ζ	10^7^ dynes×hr/cm^3^	E.E.* [23], [30]
Average cell crawling speed	V_0_	10 µm/hr	[23]
Traction stress	f_0_	10^4^ dynes/cm^2^	[30]
Dipole length	*b*	10 µm	E.E.* [30]
Rotational drag coefficient	_ζr_	400 dynes×hr/cm^2^	E.E.* [46]
Franck constant	*K*	2×10^−4^ dynes	E.E.* [26]

*E.E. (estimated from experiments. See text for more details.)

**†:** E.S. (estimated from simulations): value determined by matching simulation results to existing experimental data.

We tracked the average advance of the boundary as a function of time and compared it to data that was published previously [24] (**Figure 4**b). We find that the average border progression scales supra-linearly with time, and our simulations match the experiments by Poujade, et al. when the initial width of our simulated region was greater than 200 µm. When the initial width was smaller than 200 µm, the rate of advance of the border decreases. As in our 1D simulations, we define the border progression exponent *E* and measure the dependence of *E* on the initial width of the monolayer. For initial widths between 100–300 µm, we see an increase in the border progression exponent, which increases from 1.2 to 1.6 (**Figure 4**c). This result is also consistent with what has been measured [24].

Cell morphology and motility are known to depend on the stiffness of the substrates [38]. It is therefore interesting to ask how substrate stiffness would affect wound closure. It is likely that substrate stiffness affects the magnitude of the traction stress, the thrust force, and the resistive drag force. If all three of these parameters change in a similar fashion with respect to changes in substrate stiffness, then our model suggests that the results will be equivalent to changing the cell-cell viscosity. For example, if traction stress, thrust force, and resistive drag increase with increases in the substrate stiffness, then this would behave like decreasing the cell-cell viscosity in our model, and we would therefore only expect small changes in the overall efficiency of wound closure with changes in substrate stiffness. We also explored how wound healing would be affected by the magnitude of the dipole-distributed stress, *f*_0_, leaving all other parameters fixed. For this case, we find that wound closure is more efficient (i.e., the border progression is faster on average with larger traction stress) and the border exponent also increases. We find that increasing the traction stress by a factor of three leads to an increase in the border exponent from 1.3 to 1.8. Note that it is possible to determine the affect of changing substrate stiffness on the parameters in our model by measuring the traction stress, average speed of isolated cells, and cytoskeletal flow rates as a function of substrate stiffness, which allows a method for testing these predictions.

## Discussion

The cooperative cellular behavior that accompanies wound healing is astonishing. Even in a simple in vitro monolayer of cells, wounds are “repaired” as cellular movements fill in a denuded region. These movements have been attributed to processes such as the purse-string mechanism, where cells along the periphery of the wound concentrate actin and myosin at the wound edge. Contraction of the actin cortex by myosin can then drive wound closure, and, indeed, in chick embryos, this process is likely to play a significant role [13]; however, healing of larger wounds is presumed to rely on cell crawling. It is also possible that wounding triggers a biochemical response that signals cells to move to fill in the wound. For example, Matsubayashi et al. observed waves of phosphorylation of Mitogen Activated Protein Kinase after wounding a cell monolayer [39]. Release of reactive oxygen species were identified as potential upstream cues to these activation waves [40]. The experiments by Poujade et al. [24], which do not damage the cells during wounding, suggest that release of signaling factors at the wound site are not required for wound healing. These results suggest that a mechanical mechanism may be driving wound healing.

In this paper, we have shown that the same mechanical process that drives single cell crawling, augmented by cell-cell adhesion, is sufficient to drive wound healing. The principal driving force in this model comes from the polarization of crawling cells; i.e., single crawling cells exert a dipole-distributed force distribution on the substrate. At the edge of the wound, this force distribution acts like a pressure that pulls the cells out into the denuded region. Within the cell-filled region, the force distribution causes instabilities that lead to the observed complex flow fields of the cells, which include vortices and jets and also a fingering-like appearance of the moving boundary. Cell-cell adhesion keeps the monolayer cohesive, which produces long-range correlations in the cellular velocity field and also causes the cell monolayer to act like a viscoelastic fluid that is fairly rigid on short timescales but flows on longer timescale. This viscoelastic behavior, consequentially, produces the accelerative advance of the cells out into the wounded region. Therefore, this conceptually simple model with reasonable choices for the parameters captures quantitatively most of the observed features of wound healing.

The parameters that are used in our model are physical. Therefore, they are all, in principle, measureable. Indeed, many of the parameters have already been measured or there exist experiments that can be used to estimate the parameters. In fact, only two parameters are largely undetermined, the two effective viscosities due to cell-cell adhesions. However, the cell-cell shear viscosity in tissue has been estimated previously to be on order of 10^5^ Poise in embryonic tissue [41]. This viscosity is the three-dimensional viscosity; however, our model uses a two-dimensional that contains a factor of the thickness of the monolayer. If we assume that this thickness is around 1 µm, then our estimates based on our simulations suggest a shear viscosity of around 10^8^ Poise, which is significantly larger than the previous finding. Our results suggest, though, that the overall dynamics of wound healing are only weakly dependent on the viscosities, and, therefore, our predictions of the viscosities are probably only good to an order of magnitude.

In recent years there have been a few other models that have been proposed to describe the mechanical process of wound healing. Two separate groups have examined wound healing driven by cell proliferation and diffusive motion [42]–[44]. These models predict linear dependence of the border progression on time and do not capture the complex cellular motions that are observed in MDCK wound healing assays. However, they are able to fit well experiments using fibroblasts, which do not form adherens junctions [44]. Cellular sheets lacking adherens junctions should have a reduced viscoelastic timescale, and therefore our model would also predict a more linear advance of the border. A recent paper by Mark et al. [45] showed that treating the cells near the wound boundary as an active membrane bounding an elastic monolayer of cells can explain the fingering instability of the wound boundary. In this model, the active membrane is treated as an elastic contour that satisfies the Helfrich energy functional with surface-tension; i.e., there are energetic costs for bending and for increases in contour length. Cell migration defines an outward force at the boundary which drives the cells out into the void. Finally, there is a restoring force from the cells that are away from the boundary. It is interesting to note that the dipole-distributed traction stress in the model presented here naturally accounts for the active driving force of the Mark et al. model. It should be noted, however, that the fingering instability that we observe is not as pronounced as what is observed experimentally. In these experiments, a leader cell is often observed at the tip of the finger. These leader cells arise directly from the general population of cells and have a more-spread appearance with an active lamellipodium. Once the finger reaches the distal side of the wound, the leader cells revert back to a typical epithelial morphology. We suggest that these leader cells may be a result of reduced cadherin binding to adjacent cells; i.e., that given sufficient space to spread, that the epithelial cells will naturally spread and take on this new appearance. It is likely that a more spread cell will exert a different traction stress and thrust force than the standard cells in the population. Therefore, our model may not completely reproduce the boundary fingering because we assume that all cells in the population are equivalent.

Though the model developed here shows that it is possible that wound healing can be driven by a purely mechanical mechanism, it does not imply that cell signaling does not play an important role in this process, too. Indeed, it is definitely true that biochemical regulation is required for controlling the mechanical processes that underlie our model, specifically the actin dynamics that produce the dipole stress and thrust force, and therefore it is likely that inter-cellular signaling may modify and enhance force production in the cells that are closest to the boundary.

## Methods

### Numerical methods

The two dimensional free boundary problem was solved using the Moving Boundary Node Method [37]. This method is a level set-based, finite volume algorithm. For these simulations, we used a time step of 0.001 hr and a grid spacing that was 1/40 of the initial width of the domain (i.e., for a monolayer with an initial width of 300 µm, the grid spacing was 7.5 µm).

## Supporting Information

Text S1 Supplemental text that provides more complete details on the mathematical and computational aspects of the wound healing model presented in this paper.
(DOC)
