# Supplementary material for: Crawling Cells Can Close Wounds without Purse Strings or Signaling
Source: PLoS Comput Biol. 2011 Mar 10;7(3):e1002007. doi: 10.1371/journal.pcbi.1002007 (PMC3053312; doi:10.1371/journal.pcbi.1002007)
Supplement: Text S1 — Supplemental text that provides more complete details on the mathematical and computational aspects of the wound healing model presented in this paper. (DOC) [file pcbi.1002007.s001.doc]

# Supplementary Information Appendix

# S1. Force balance in epithelial monolayers during wound healing

# In this section, we derive the mathematical model for wound healing that we present in this paper. We begin by considering the stresses that act on a single crawling cell. Then, force balance between neighboring cells is used to develop a mean-field description of the dynamics of the epithelial cell monolayer.

# The inside of a single crawling cell can be considered to be a continuous medium; i.e., if we consider length scales larger than the constituent molecules but smaller than the cell, then we can define averaged quantities that describe the local stress, velocity, etc. In general, at the cellular scale, resistive drag forces dominate over inertia. Therefore, the sum of all the forces on any small region inside the cell must be equal to zero. Since external forces can typically only be applied at the boundary of the cell, Newton’s third law demands that the force exerted on a small, internal region of the cell, must be equal and opposite to the force that the small region exerts onto the neighboring regions. Therefore, we can define the internal stress tensor σ_in_, which describes the force per area that acts on the surface of our subregion. In the limit that the size of this region goes to zero, it must be true that

$\nabla\cdot\sigma_{in}=0$ (A1)

# At the surface of the cell, the normal component of the stress is equal to the applied force per area, $\sigma_{in}\cdot\mathbf{n}=\mathbf{f}$. For cells in contact with a substrate, it is typical to assume that the force per area is proportional to the cytoskeletal velocity (see, for example, (1, 2). Since most surface-bound cells are well-spread, the height of the cell is small compared to the area that covers the substrate. Therefore, it is often useful to consider the dynamics of a thin moving cell, by averaging over the height dimension (i.e., the dimension that is perpendicular to substrate). As shown in (2), under this approximation, Eq. A1 becomes

$\nabla\cdot\sigma_{in}^{2D}=\zeta\mathbf{v}$, (A2)

# where σ_in_^2D^ is the stress in the plane parallel to the substrate, ζ is a drag coefficient, and v is the velocity of the cytoskeleton with respect to the substrate.

# The cytoskeletal velocity is spatially-dependent. Most crawling cells have a retrograde flow of actin at the leading edge, and the cytoskeleton at the rear of the cell moves with the velocity of the cell. Therefore, we decompose the cytoskeletal velocity using the net cell velocity V_0_ and a deviation velocity δv; v = V_0_ + δv. We assume that the velocity of the cell is in the direction of the polarization of the cell, V_0_ = V_0_d. The thrust force that the cell exerts on the substrate is F = ζV_0_*A*d = *A*f, where *A* is the area of the cell, and f is the force per area.

# From our definitions, the deviation velocity δv is zero at the rear of the cell and negative at the front of the cell. The internal cellular stress must generate this flow. In general, we can write the internal stress as a function of position as

$\sigma_{in}^{2D}=f\left(x,y\right)\hat{\mathbf{I}}+g\left(x,y\right)\mathbf{dd}+h\left(x,y\right)\mathbf{de}$, (A3)

# where $\hat{\mathbf{I}}$is the identity matrix, and e is the unit vector perpendicular to d. For highly polarized cells, we choose set f(x,y) and h(x,y) equal to zero. In addition, in the absence of other external forces applied to the cell, the two-dimensional internal stress must equal zero at the edges of the cell. These conditions imply that the average internal stress is a positive number, $\left\langle\sigma_{in}^{2D}\right\rangle=f_{0}b\mathbf{dd}$, where f_0_ is a positive number that represents the magnitude of the force per area that the cell exerts against the substrate, and *b* is the length of the cell. From our definitions, the deviation velocity is

$\delta\mathbf{v}=\frac{1}{\zeta}\nabla\cdot\sigma_{in}^{2D}-V_{0}\mathbf{d}$. (A4)

# We can now use these results to write an equation for the force balance inside an epithelial cell monolayer. Treating the monolayer as a continuous medium, we define the stress σ_m_ in the monolayer. Averaging this stress over the height of the cell layer and assuming that the substrate produces a resistive drag against the cells, the equation for the monolayer is analogous to (Eq. A2):

$\nabla\cdot\sigma_{m}=\zeta\mathbf{v}$, (A5)

# where v is the local velocity of the actin flow inside the monolayer. Once again, we decompose this velocity into an average velocity of the cells centered at the location x, V(x), and the internal variations in the cytoskeletal velocities, dv. If we assume that the intracellular cytoskeletal flows remain roughly the same, then we can re-write Eq. A5 as

$\nabla\cdot\sigma_{m}=\zeta\mathbf{V}+\nabla\cdot\sigma_{in}^{2D}-\mathbf{F}$ (A6)

# which is equivalent to Eq. 2 in the main text, when the dipole stress is taken to be s_d_ = -f_0_*b*dd.

A constitutive relation is required to define the monolayer stress. For this, we assume that forces exerted between cells are withstood by cadherin adhesions. Since cadherin molecules turnover, the cells can slip with respect to each other. As we show below, this dynamics can be approximated by the Maxwell model for viscoelastic fluids.

# S2. The viscoelasticity of cell-cell adhesions

Cell-cell adhesion is mediated by cadherin molecules (3, 4). Fluorescent recovery after photobleaching (FRAP) experiments show that cadherin turnover in adhesions has a characteristic rate of around 3.9 hr^-1^ (4). Therefore, we expect that on timescales less than 15 minutes, that nearby cells remain well adhered, and that the cell monolayer will behave elastically for times less than this. However, for times longer than 15 minutes, the adhesions can break, and the cells can slip. Therefore, we expect more viscous behavior. Indeed, a one-dimensional model of a block coated with proteins that moves along a substrate with time dependent velocity *v*(t), where the interaction between the block and the substrate is mediated solely by binding and stretching of the proteins, leads to complex rheological behavior. For example, consider that proteins can only bind in an unstretched state with a rate constant *k*_on_. Motion of the block then stretches the proteins. How stretched the protein depends on how far the block has moved since the protein bound. At any given time, some proteins will be more stretched than others. The concentration of proteins at time t that bound to the substrate at time t - τ can be defined as C_b_(t,τ). If there is a constant off-rate, *k*_off_, then the kinetic equation for C_b_ is

$\frac{\partial C_{b}}{\partial t}+\frac{\partial C_{b}}{\partial\tau}=k_{on}C_{u}\delta\left(\tau\right)-k_{off}C_{b}$,

where C_u_ is the concentration of unbound proteins. This model is similar to the Lacker-Peskin model for muscle with an added advective term (5). Assuming the bound proteins behave like linear springs with spring constant Λ, the force per area (the stress, σ) on the cell is

$\sigma=\frac{F}{A}=-\Lambda\int_{0}^{\infty} C_{b}\left(\int_{t-\tau}^{t} v\left(t'\right)dt'\right)d\tau$,

Taking the time derivative of Eq. (B2), it can be shown that the stress behaves like the linear Maxwell model (6),

$$\sigma+k_{_{off}}^{-1}\frac{\partial\sigma}{\partial t}=2\Lambda v\int_{-\infty}^{0} C_{b}d\tau$$

For cells that are sliding against one another, the velocity in Eq. (B3) represents the velocity difference between the cells. Therefore, in a continuum limit, the righthand side of Eq. (B3) is proportional to the rate of strain tensor. This analysis justifies the use of the linear Maxwell model to describe the cell-cell interaction stress,

Where *k* is comparable to the FRAP recovery time for cadherin, η is a viscosity that depends on the cadherin bond strength, and is the rate of strain tensor. Using a viscous fluid model, the cell-cell viscosity has been estimated (7).

The formulation shown in Eq. B4 treats sliding (shear) deformations on an equal footing with deformations that change the density of the cells in the monolayer. Since cells can slide on top of each other, we imagine that changes in density are possible, and that they are resisted in a similar fashion to shear deformations. However, we assume that the effective viscosity for volumetric changes is larger than the shear viscosity. Therefore, we define a volumetric viscosity coefficient λ to take into account this difference. The resulting equation is Eq. 1 in the main text.

**S3. Re-orientation by flow**

If a rigid rod is placed in a spatially non-uniform flow, the rod will re-orient. To calculate the torque on the rod due to the flow, we assume that the force on any small segment of the rod is proportional to the velocity, with the drag coefficient ζ defining the constant of proportionality. If the orientation of the rod is along the direction **d**, the torque about the center of mass is then

$\mathbf{T}=\int_{-b/2}^{b/2} \zeta s\mathbf{d}\times\mathbf{v}\left(\mathbf{x}+s\mathbf{d}\right)ds$,

where b is the length of the rod and s is the distance from the center of mass to a point on the rod. If the variation in the velocity is small on lengths comparable to the rod length, then we can make a Taylor series expansion of the velocity about the center of mass of the rod. Keeping terms up to first order in b, we find

$$\begin{matrix} \mathbf{T}\approx\int_{-b/2}^{b/2} \zeta s\mathbf{d}\times\left(\mathbf{v}+s\left(\mathbf{d}\cdot\nabla\right)\mathbf{v}\right)ds \\ \approx\frac{\zeta b^{3}}{12}\mathbf{d}\times\left(\left(\mathbf{d}\cdot\nabla\right)\mathbf{v}\right) \end{matrix}$$

Eq. C.2 is the torque on the rod due to the flow (this is the same torque that is derived in Chapter 8 of (8)). Since we are working in two dimensions, we need the torque per area. If we assume that the area of the rod is b^2^, then the torque per area that is due to the flow is ζb(**d×**(**d**·)**v**)/12. In two dimensions, if we write that **d** = cosθ**x** + sinθ**y**, then the torque per length is

$\frac{\zeta b}{24}\left(\left(\frac{\partial v_{y}}{\partial x}-\frac{\partial v_{x}}{\partial y}\right)+cos2\theta\left(\frac{\partial v_{y}}{\partial x}+\frac{\partial v_{x}}{\partial y}\right)+sin2\theta\left(\frac{\partial v_{y}}{\partial y}-\frac{\partial v_{x}}{\partial x}\right)\right)\mathbf{z}$.

The first term in C.3 is the vorticity, which describes the total local angular velocity of the fluid. The other two terms depend on the orientation of the rod with respect to the gradient of the velocity. In fact, the second term in this equation arises due to the fact that we have considered the torque on a very thin object. A circular-shaped cell would not include this term. The third term in this equation describes reorientation by convergent (or divergent) flow. We imagine that a cell is more likely to compress than to reorient due to these types of flow. Therefore, we only consider re-orientation due to the vorticity (i.e., the first term in this equation). Under this assumption, the torque from the fluid flow causes a reorientation of the alignment vector given by

$$\frac{\partial\mathbf{d}}{\partial t}=\frac{1}{2}\left(\nabla\times\mathbf{v}\right)\times\mathbf{d}$$

Including advection and elastic reorientation to the alignment dynamics leads to Eq. 3 in the main text.

**S4. Derivation of the averaged one-dimensional model**

If we consider an infinite strip of cells aligned with the *y*-axis and average the dynamic equations over the *y* coordinate, under the assumption that the cellular orientation is randomly distributed, then the average value of the orientation is zero. We can therefore assume that the average quantities of the monolayer stress tensor and the cell velocity do not depend on *y*:

$$v\equiv v(x),\text{ }\hat{\sigma}_{ij}\equiv\hat{\sigma}_{ij}(x)$$

Since the orientation field $d=\left(\cos\theta,\sin\theta\right)$ is uniformly distributed with $\theta\in[-\pi,\pi)$, the integral of the dipole stress produces a constant pressure term:

$$<b\hat{d}\hat{d}>_{\theta}=\frac{1}{2}b\left(\hat{x}\hat{x}+\hat{y}\hat{y}\right)$$

The force balance equation is then greatly simplified, with the velocity being proportional to gradients in the stress:

$$\zeta v_{x}=\frac{\partial\sigma_{xx}}{\partial x}+\frac{\partial\sigma_{xy}}{\partial y}=\frac{\partial\sigma_{xx}}{\partial x}$$

Inserting Eq. D3 into the viscoelastic equation for the cell-cell adhesion dynamics, we get a reaction-diffusion equation for the stress:

$\tau_{VE}\frac{\partial\sigma_{xx}}{\partial t}+\sigma_{xx}=\left(\lambda+\frac{\eta}{2}\right)\frac{\partial v_{x}}{\partial x}=\frac{\left(\lambda+\frac{\eta}{2}\right)}{\zeta}\frac{\partial^{2}\sigma_{xx}}{\partial x^{2}}$.

In the absence of other forces, this equation is subject to the boundary condition that the total stress (cell-cell adhesion stress plus the dipole stress) at the boundary is zero:

$\hat{x}\cdot\left(\hat{\sigma}-f_{0}b\hat{d}\hat{d}\right)\cdot\hat{x}=0$.

Therefore,

$\left.\sigma_{xx}\right|_{boundary}=\frac{f_{0}b}{2}$.

To gain some insight into the expected behavior of this system, we consider the limit where the cadherin timescale is small (which is also the same as considering the long time behavior of the system). Setting τ to zero, we obtain the following formula on the stress distribution and the velocity at the border:

$$\begin{matrix} \sigma=\frac{f_{0}b}{2\cosh\frac{kL}{2}}(\cosh kx),\text{ }k^{2}=\frac{\zeta}{\left(\lambda+\eta/2\right)} \\ V_{L/2}=\frac{f_{0}b}{2\sqrt{\zeta\left(\lambda+\eta/2\right)}}\tanh(\sqrt{\frac{\zeta}{\left(\lambda+\eta/2\right)}}\frac{L}{2}) \end{matrix}$$

For small L, the velocity at the border $V_{L/2}$ is proportional to the elapsed time, and for large $L$, it gets close to a constant. Therefore, the wound healing progress scales like time-squared at short times and is linear with time for long times.

**S5. Derivation of the equations governing the closure of circularly-symmetric wounds**

For a circular-shaped wound, we can consider the behavior of the model system (Eqs. 1-3, in the main text) in polar coordinates. If we assume that the dynamics are circularly-symmetric (i.e., that the variables only depend on the radial coordinate *r* and are independent of the azimuthal direction), then we can construct a simplified, one-dimensional model for the average dynamics. Under this assumption, the cellular orientation field obeys the following dynamic equation:

$\left\{\begin{matrix} \frac{\partial d_{r}}{\partial t}+\tilde{v}_{r}\frac{\partial d_{r}}{\partial r}=-\frac{1}{2}\frac{\partial\tilde{v}_{\theta}}{\partial r}d_{\theta}+\frac{2\tilde{K}}{r}\frac{\partial d_{r}}{\partial r}+\tilde{K}\frac{\partial^{2}d_{r}}{\partial r^{2}} \\ \frac{\partial d_{\theta}}{\partial t}+\tilde{v}_{r}\frac{\partial d_{\theta}}{\partial r}=\frac{1}{2}\frac{\partial\tilde{v}_{\theta}}{\partial r}d_{r}+\frac{2\tilde{K}}{r}\frac{\partial d_{\theta}}{\partial r}+\tilde{K}\frac{\partial^{2}d_{\theta}}{\partial r^{2}} \end{matrix}\right.$ (E.1)

and the time derivative of the stress tensor is given by:

$\left\{\begin{matrix} \tilde{\sigma}_{rr}+\tilde{\tau}\frac{\partial\tilde{\sigma}_{rr}}{\partial t}=\tilde{\eta}\frac{\partial\tilde{v}_{r}}{\partial r}\text{ } \\ \tilde{\sigma}_{r\theta}+\tilde{\tau}\frac{\partial\tilde{\sigma}_{r\theta}}{\partial t}=\frac{\tilde{\eta}}{2}(\frac{\partial\tilde{v}_{\theta}}{\partial r}-\frac{\tilde{v}_{\theta}}{r}) \end{matrix}\right.$ (E.2)

where tilde denotes dimensionless variables.

In the active cell zone (Zone A), we apply the force balance relation (Eq. 2) with the dipole stress and thrust force terms included. Since inhibition of Rac reduces actin dynamics, we omit the thrust force and dipole stress terms in the deactivated cell zone (Zone B). The force balance equations in these two regions are therefore given by:

$(A)\text{ }\left\{\begin{matrix} \tilde{v}_{r}=d_{r}-\tilde{b}(\frac{\partial d_{r}}{\partial r}+\frac{d_{r}}{r})d_{r}+\frac{\partial\tilde{\sigma}_{rr}}{\partial r}+\frac{\tilde{\sigma}_{rr}}{r} \\ \tilde{v}_{\theta}=d_{\theta}-\tilde{b}(\frac{\partial d_{r}}{\partial r}+\frac{d_{r}}{r})d_{\theta}+\frac{\partial\tilde{\sigma}_{r\theta}}{\partial r}+\frac{\tilde{\sigma}_{r\theta}}{r} \end{matrix}\right.\text{ (B) }\left\{\begin{matrix} \tilde{v}_{r}=\frac{\partial\tilde{\sigma}_{rr}}{\partial r}+\frac{\tilde{\sigma}_{rr}}{r} \\ \tilde{v}_{\theta}=\frac{\partial\tilde{\sigma}_{r\theta}}{\partial r}+\frac{\tilde{\sigma}_{r\theta}}{r} \end{matrix}\right.$ (E.3)

At the boundary between these two zones, continuity of the total stress implies that

$(\left.\tilde{\sigma}\right|_{\text{living}}-\left.\tilde{\sigma}\right|_{\text{dead}})\cdot r=\tilde{b}\hat{d}\hat{d}\cdot r$, (E.4)

and at the wound boundary,

$\left.\tilde{\sigma}\right|_{\text{dead}}\cdot r=0$. (E.5)

**S6. Numerical method**

The procedure that we use to solve the dynamical equations for the collective cell migration model is described on 1-D and 2-D.

**Averaged 1-D model**

At the time step $t^{n}$, the spatial domain spans from –*L^n^*/2 to *L^n^*/2, with $L^{n}$ the width of the tissue. Because the equations are symmetric with respect to x=0, we only consider the domain from 0 to *L^n^*/2, with the boundary condition for stress, $\frac{\partial\sigma}{\partial x}=0$ at $x=0$. The discrete coordinates are uniformly distributed as $x_{i}^{n}=i\Delta x^{n}$ with $\Delta x^{n}=\frac{L^{n}/2}{N}$.

The governing equation of (C4) is discretized as follows:

$$\tau_{VE}\frac{\sigma_{i}^{*}-\sigma_{i}^{n}}{\Delta t}+\sigma_{i}^{*}=\frac{(\lambda+\frac{\eta}{2})}{\xi}\frac{\sigma_{i+1}^{*}-2\sigma_{i}^{*}+\sigma_{i-1}^{*}}{(\Delta x^{n})^{2}}$$

From this implicit time-stepping, the intermediate stress $\sigma_{i}^{*}$ is obtained. Using the relationship between the migration speed and the stress gradient of Eq. (D3), the domain length $L^{n}$ is updated by the following:

$L^{n+1}=L^{n}+(\frac{f_{0}b}{2}-\sigma_{N-1}^{*})/\Delta x^{n}$.

The new coordinates $x_{i}^{n+1}$ are then redistributed uniformly with the grid size $\frac{L^{n+1}/2}{N}$. The stress $\sigma_{i}^{n+1}=\sigma^{n+1}(x_{i}^{n+1})$ on the updated domain $[0\text{ }\frac{L^{n\text{+}1}}{2}]$ is computed by the piecewise cubic spline interpolation from $\sigma_{i}^{*}=\sigma^{*}(x_{i}^{n})$. Note that while the domain size is changing, the total grid number is the same as N.

**The closure of circularly-symmetric wounds**

The time-stepping from $t^{n}$ to $t^{n+1}$ is done sequentially updating the orientation, the stress tensor, and then the velocity as follows:

I ) $\begin{matrix} \frac{d_{r,i}^{n+1}-d_{r,i}^{n}}{\Delta t}+\tilde{v}_{i}^{\text{UPWIND}}\frac{d_{r,i+1}^{n+1}-d_{r,i}^{n+1}}{\Delta r^{n}}=-\frac{1}{2}\frac{\tilde{v}_{\theta,i+1}^{n}-\tilde{v}_{\theta,i}^{n}}{\Delta r^{n}}d_{\theta,i}^{n}+\tilde{K}\frac{d_{r,i+1}^{n+1}-2d_{r,i}^{n+1}+d_{r,i-1}^{n+1}}{(\Delta r^{n})^{2}} \\ \frac{d_{\theta,i}^{n+1}-d_{\theta,i}^{n}}{\Delta t}+\tilde{v}_{i}^{\text{UPWIND}}\frac{d_{\theta,i+1}^{n+1}-d_{\theta,i}^{n+1}}{\Delta r^{n}}=\frac{1}{2}\frac{\tilde{v}_{\theta,i+1}^{n}-\tilde{v}_{\theta,i}^{n}}{\Delta r^{n}}d_{r,i}^{n}+\tilde{K}\frac{d_{\theta,i+1}^{n+1}-2d_{\theta,i}^{n+1}+d_{\theta,i-1}^{n+1}}{(\Delta r^{n})^{2}} \end{matrix}$

where $v_{i}^{\text{UPWIND}}$ is determined dependent on the direction of the effective advection velocity:

$$\tilde{v}_{i}^{\text{UPWIND}}=\left\{\begin{matrix} \frac{1}{2}(\tilde{v}_{r,i-1}^{n}+\tilde{v}_{r,i}^{n})-\frac{2\tilde{K}}{r_{i-1/2}},\text{ }\tilde{v}_{r,i}^{n}\geq\frac{2\tilde{K}}{r_{i}}\text{ } \\ \frac{1}{2}(\tilde{v}_{r,i}^{n}+\tilde{v}_{r,i+1}^{n})-\frac{2\tilde{K}}{r_{i+1/2}},\text{ }\tilde{v}_{r,i}^{n}\text{<}\frac{2\tilde{K}}{r_{i}} \end{matrix}\right.$$

II ) $\begin{matrix} \tilde{\sigma}_{rr,i}^{n+1}+\tilde{\tau}\frac{\tilde{\sigma}_{rr,i}^{n+1}-\tilde{\sigma}_{rr,i}^{n}}{\Delta t}=\tilde{\eta}\frac{\tilde{v}_{r,i+1}^{n}-\tilde{v}_{r,i-1}^{n}}{2\Delta r^{n}} \\ \tilde{\sigma}_{r\theta,i}^{n+1}+\tilde{\tau}\frac{\tilde{\sigma}_{r\theta,i}^{n+1}-\tilde{\sigma}_{r\theta,i}^{n}}{\Delta t}=\frac{\tilde{\eta}}{2}(\frac{\tilde{v}_{\theta,i+1}^{n}-\tilde{v}_{\theta,i-1}^{n}}{2\Delta r^{n}}-\frac{\tilde{v}_{\theta,i}^{n}}{r_{i}^{n}}) \end{matrix}$

The discretization of the gradient of the velocity is accurate in the second order.

IIIA) $\begin{matrix} \tilde{v}_{r,i}^{n}=d_{r,i}^{n}-\tilde{b}(\frac{d_{r,i+1}^{n}-d_{r,i-1}^{n}}{2\Delta r^{n}}+\frac{d_{r,i}^{n}}{r_{i}^{n}})d_{r,i}^{n}+\frac{\tilde{\sigma}_{rr,i+1}^{n}-\tilde{\sigma}_{rr,i-1}^{n}}{2\Delta r^{n}}+\frac{\tilde{\sigma}_{rr,i}^{n}}{r_{i}^{n}} \\ \tilde{v}_{\theta,i}^{n}=d_{\theta,i}^{n}-\tilde{b}(\frac{d_{r,i+1}^{n}-d_{r,i-1}^{n}}{2\Delta r^{n}}+\frac{d_{r,i}^{n}}{r_{i}^{n}})d_{\theta,i}^{n}+\frac{\tilde{\sigma}_{r\theta,i+1}^{n}-\tilde{\sigma}_{r\theta,i-1}^{n}}{2\Delta r^{n}}+\frac{\tilde{\sigma}_{r\theta,i}^{n}}{r_{i}^{n}} \end{matrix}$

In the domain of the dead cell zone, the migrating velocity equation is simplified as follows:

IIIB) $\begin{matrix} \tilde{v}_{r,i}^{n}=\frac{\tilde{\sigma}_{rr,i+1}^{n}-\tilde{\sigma}_{rr,i-1}^{n}}{2\Delta r^{n}}+\frac{\tilde{\sigma}_{rr,i}^{n}}{r_{i}^{n}} \\ \tilde{v}_{\theta,i}^{n}=\frac{\tilde{\sigma}_{r\theta,i+1}^{n}-\tilde{\sigma}_{r\theta,i-1}^{n}}{2\Delta r^{n}}+\frac{\tilde{\sigma}_{r\theta,i}^{n}}{r_{i}^{n}} \end{matrix}$

The discretization of the gradient of the orientation and stress is also of the second order. For the case without a dead cell zone, a unified computational domain $D_{\text{L}}=[r_{b}\text{ }r_{\text{E}}]$ is used with $r_{b}$ the coordinate of the wound boundary moving, and $r_{\text{E}}$ the coordinate of the outer boundary fixed. When the dead cell zone is considered, two subdomains $D_{\text{D}}=[r_{b}\text{ }r_{i}]$ and $D_{\text{L}}=[r_{i}\text{ }r_{\text{E}}]$ are used with $r_{i}$ the coordinate of the interface between dead and living cell zones. When we treat the two subdomains together, the time-stepping of I), II), IIIA) are done first for the living cell zone of $D_{\text{L}}$, and then the time-stepping of I), II), IIIB) are taken. The cross-talk between the two subdomains are bridged based on Eq. (E4):

$$\begin{matrix} \left.\tilde{\sigma}_{rr}^{n}\right|_{r_{i}}^{D_{\text{D}}}=\left.\tilde{\sigma}_{rr}^{n}\right|_{r_{i}}^{D_{\text{L}}}-\tilde{b}(\left.d_{r}\right|_{r_{i}}^{D_{\text{L}}})^{2} \\ \left.\tilde{\sigma}_{r\theta}^{n}\right|_{r_{i}}^{D_{\text{D}}}=\left.\tilde{\sigma}_{r\theta}^{n}\right|_{r_{i}}^{D_{\text{L}}}-\tilde{b}(\left.d_{r}\right|_{r_{i}}^{D_{\text{L}}})(\left.d_{\theta}\right|_{r_{i}}^{D_{\text{L}}}) \end{matrix}$$

with the jump term from the dipole stress. We assume that the orientation $(d_{r},d_{\theta})$ and velocity$(v_{r},v_{\theta})$, are continuous at the interface.

**Two dimensional dynamics of a wounded epithelial monolayer**

The initial cell orientation is distributed randomly with the uniform distribution in orientation angle. To place random cellular polarization at the initial time, we assume the initial orientation $\theta\in[-\pi,\pi)$.

The whole time-stepping is composed of the 4 procedures:

STEP 1: Each time step is determined to take movement less than the spatial grid size in each time-stepping.

STEP 2: Take step on the cellular orientation dynamics with the torque free orientation at the boundary.

STEP 3: Take step on cellular stress dynamics with the dipole stress boundary condition.

STEP 4: Take step on border progress.

For simplicity, we apply the following notations for the advection and curl operators $A^{n}$ and $\omega^{n}$ with the finite volume $\Delta V^{n}$ at time step $n$:

$$\begin{matrix} A^{n}=\Delta V^{n}v^{n}(\nabla\cdot)^{n} \\ \omega^{n}=\nabla^{n}\times v^{n} \end{matrix}$$

While time-stepping from $t^{n}$ to $t^{n+1}$, we incorporate the Crank-Nickolson second order method, which is described in the sub-steps (A) and (B).

**A. Intermediate time stepping**: $(\tilde{v}^{n},d^{n},\tilde{\sigma}^{n},\varphi^{n})\to(\tilde{v}^{n+1/2},d^{n+1/2},\tilde{\sigma}^{n+1/2},\varphi^{n+1/2})$

$$\begin{matrix} 1A)\text{ }(\frac{2\Delta V^{n+1/2}}{\Delta t}I-\tilde{K}L^{n+1/2})d^{n+1/2}=\frac{2\Delta V^{n}}{\Delta t}d^{n}-A^{n}d^{n}+\frac{1}{2}\omega^{n}\times d^{n}\Delta V^{n} \\ 1B)\text{ (}\Delta V^{n+1/2}+\frac{2\tilde{\tau}\Delta V^{n+1/2}}{\Delta t})\tilde{\sigma}^{n+1/2}=\frac{2\tilde{\tau}\Delta V^{n}}{\Delta t}\tilde{\sigma}^{n}+\frac{\tilde{\eta}}{2}(D^{n}\tilde{v}^{n}+(D^{n}\tilde{v}^{n})^{T}) \\ 1C)\text{ }\Delta V^{n+1/2}\tilde{v}^{n+1/2}=D^{n+1/2}\cdot\tilde{\sigma}^{n+1/2}-\tilde{b}D^{n+1/2}\cdot(d^{n+1/2}d^{n+1/2})+d^{n+1/2}\Delta V^{n+1/2} \end{matrix}$$

**B. Full time stepping**: $(\tilde{v}^{n},d^{n},\tilde{\sigma}^{n},\varphi^{n},\tilde{v}^{n+1/2},d^{n+1/2},\tilde{\sigma}^{n+1/2},\varphi^{n+1/2})\to(\tilde{v}^{n+1},d^{n+1},\tilde{\sigma}^{n+1},\varphi^{n+1})$

$$\begin{matrix} 2A)\text{ }(\frac{\Delta V^{n+1}}{\Delta t}I-\frac{1}{2}\tilde{K}L^{n+1})d^{n+1} \\ \text{ }=\frac{\Delta V^{n}}{\Delta t}d^{n}-A^{n+1/2}d^{n+1/2}+\frac{1}{2}\omega^{n+1/2}\times d^{n+1/2}\Delta V^{n+1/2} \\ 2B)\text{ (}\frac{\Delta V^{n+1/2}}{2}+\frac{\tilde{\tau}\Delta V^{n+1/2}}{\Delta t})\tilde{\sigma}^{*} \\ \text{ }=(-\frac{\Delta V^{n}}{2}+\frac{\tilde{\tau}\Delta V^{n}}{\Delta t})\tilde{\sigma}^{n}+\frac{\tilde{\eta}}{2}(D^{n+1/2}\tilde{v}^{n+1/2}+(D^{n+1/2}\tilde{v}^{n+1/2})^{T}) \end{matrix}$$

Regarding the prescription on the stress boundary condition, we apply the left-handed and right-handed projections of the intermediate boundary stress tensor $\tilde{\sigma}_{_{\text{VE}}}^{*}$in the tangential direction $t$ and $n$ as follows:

$$\begin{matrix} n\cdot\tilde{\sigma}^{n+1}\cdot n=n\cdot(\tilde{b}nn)\cdot n \\ t\cdot\tilde{\sigma}^{n+1}\cdot n=t\cdot(\tilde{b}nn)\cdot n \\ t\cdot\tilde{\sigma}^{n+1}\cdot t=t\cdot\tilde{\sigma}^{*}\cdot t \end{matrix}$$

After algebraic rearrangement,

$$P\tilde{\sigma}^{n+1}=\tilde{\sigma}^{P}$$

where

$$\tilde{\sigma}^{P}=\left(\begin{matrix} t\cdot\tilde{\sigma}^{*}\cdot t \\ 0 \\ \tilde{b} \end{matrix}\right)$$

and the projection matrix is constituted in the following:

$$P=\left(\begin{matrix} n_{y}^{2} & -2n_{x}n_{y} & n_{x}^{2} \\ n_{x}^{2} & 2n_{x}n_{y} & n_{y}^{2} \\ n_{x}n_{y} & n_{x}^{2}-n_{y}^{2} & n_{x}n_{y} \end{matrix}\right)$$

The determination on the migrating velocity is done with the projected stress$\sigma_{_{\text{VE}}}^{n+1}$

$2C)\text{ }\Delta V^{n+1}\tilde{v}^{n+1}=D^{n+1}\cdot\tilde{\sigma}^{n+1}-\tilde{b}D^{n+1}\cdot(d^{n+1}d^{n+1})+d^{n+1}\Delta V^{n+1}$.

**References**

1. Rubinstein B, Fournier MF, Jacobson K, Verkhovsky AB, & Mogilner A (2009) Actin-myosin viscoelastic flow in the keratocyte lamellipod. *Biophys. J.* 97:1853-1863.

2. Zajac M, Dacanay B, Mohler WA, & Wolgemuth CW (2008) Depolymerization-driven flow in nematode spermatozoa relates crawling speed to size and shape. *Biophys. J.* 94:3810-3823.

3. Chen CP, Posy S, Ben-Shaul A, Shapiro L, & Honig BH (2005) Specificity of cell-cell adhesion by classical cadherins: Critical role for low affinity dimerization through beta-strand swapping. *Proc. Natl. Acad. Sci. USA* 102:8531-8536.

4. Lambert M*, et al.* (2007) Nucleation and growth of cadherin adhesions. *Exp. Cell Res.* 313:4025-4040.

5. Lacker HM & Peskin CS (1986) A mathematical method for the unique determination of cross-bridge properties from steady-state mechanical and energetic experiments on macroscopic muscle. *Lectures on mathematics in the life sciences.* 16:121-153.

6. Morrison FA (2001) *Understanding rheology* (Oxford University Press, Oxford).

7. Beysens DA, Forgacs G, & Glazier JA (2000) Cell sorting is analogous to phase ordering in fluids. *Proc. Natl. Acad. Sci. USA* 97:9467-9471.

8. Doi M & Edwards SF (1986) *The Theory of Polymer Dynamics* (Oxford University Press, Oxford).
